# Assessment of health care needs and utilization in a mixed public-private system: the case of the Athens area

**DOI:** 10.1186/1472-6963-6-146

**Published:** 2006-11-02

**Authors:** Evelina Pappa, Dimitris Niakas

**Affiliations:** 1 Faculty of Social Sciences, Hellenic Open University, Patras, Greece

## Abstract

**Background:**

Given the public-private mix of the Greek health system, the purpose of this study was to assess whether variations in the utilisation of health services, both primary and inpatient care, were associated with underlying health care needs and/or various socio-economic factors.

**Methods:**

Data was obtained from a representative sample (N = 1426) residing in the broader Athens area (response rate 70.6%). Perceived health-related quality of life (HRQOL), as measured by the physical and mental summary component scores of the SF-36 Health Survey, was used as a proxy of health care need. Health care utilization was measured by a) last-month visits to public sector physicians, b) last-month visits to private sector physicians, c) last-year visits to hospital emergency departments and d) last-year hospital admissions. Statistical analysis involved the implementation of logistic regression models.

**Results:**

Health care need was the factor most strongly associated with all measures of health care utilization, except for visits to public physicians. Women, elderly, less wealthy and individuals of lower physical health status visited physicians contracted to their insurance fund (public sector). Women, well educated and those once again of lower physical health status were more likely to visit private providers. Visits to hospital emergency departments and hospital admissions were related to need and no socio-economic factor was related to the use of those types of care.

**Conclusion:**

This study has demonstrated a positive relationship between health care need and utilisation of health services within a mixed public-private health care system. Concurrently, interesting differences are evident in the utilization of various types of services. The results have potential implications in health policy-making and particularly in the proper allocation of scarce health resources.

## Background

Several studies have identified perceived health status or health-related quality of life (HRQOL) as a very important predictor of health service utilization [1-7]. Furthermore, many studies provide evidence that demographic characteristics also affect health care need and utilization. Specifically, women report poorer health and use primary health services more often than men, have higher rates of hospitalisation and surgery and increased total expenditures, whereas men are less likely to receive preventive medical services [3,6,8,9]. Age is positively related to hospital admissions [7], and also to an above average length of stay, increased utilization of services and, overall, to greater need [10]. Regarding men, marital status and specifically married, in a de facto relationship or divorced, was found to be positively associated with a first or further visits to a GP [3,4].

Other studies have focused on the extent to which socio-economic differences affect the use of health services. Individuals with a lower level of education make fewer visits to specialists [11,12]. Contrarily, those with a higher education or income, after adjustment for socio-demographic and need variables [1,4,13], demonstrate an increased likelihood of accessing GP services or making at least one visit to a specialist. Morris [2] reports a pro-rich inequality in all types of hospital care and a pro-poor inequality in GP visits. Furthermore, an inverse income relationship has been observed with respect to needed, but not sought, medical care, with the proportion of individuals not seeking needed medical care increasing significantly in lower income groups [14]. On the other hand, visit delay and/or cancellation, and underutilization of physician services were more evident in younger individuals, low-income and economically troubled, in chronic medical conditions or in individuals without a regular source of care or a physical care discount card [15].

Many studies have examined the influence, on service utilization, of factors such as race/ethnicity or community area (rural/urban), with the latter differentiating access in favour of urban area residents [16]. In the UK, it has been shown that Indians use more GP services than other minorities. Moreover children and young people from all minority ethnic groups make lower use of outpatient and inpatient services compared to white children and young people, and these differences persisted after controlling for socio-economic and health status variables [17,18].

The purpose of this study was to examine if demographic, socio-economic and need characteristics of individuals influenced their use of the following health services: visits to public sector physicians, visits to private sector physicians, visits to hospital emergency outpatient departments and admissions to hospitals. In addition we attempted to identify factors that differentiate the use of public and private services. Based on previous research it was hypothesized that need factors ranked as the most important determinant of health care use. Concerning the different use of public and private services, it was assumed that socio-economic factors played an important role. This study contributes to the available literature as it investigates variations in health care service utilization in Greece, by using SF-36 Summary Scores, instead of the usual eight subscales, as proxies of health care needs. The results have potential health policy and planning implications and could support administrators in their difficult task of properly allocating health resources.

### Perceived health as a measure of health care need

Self-assessed health status was measured with *the SF-36 Health Survey*, a generic instrument constructed to be a brief alternative in health policy evaluations, general population surveys, clinical research and practice. It is the most widely used measure of self-perceived health with the aim of assessing concepts representing basic human values relevant to health status and well-being. The SF-36 has proven useful in comparing general and specific populations and for assessing the impact of disease and treatments on patients' perceived health state and quality of life [19]. The SF-36 is a multi-item questionnaire comprised of eight scales: Physical Functioning (PF), Role limitations due to Physical problems (RP), Bodily Pain (BP), General Health (GH), Vitality (VT), Social Functioning (SF), Role limitations due to Emotional problems (RE) and Mental Health (MH) [19].

Factor analytic studies have shown that the physical and mental summary factors account for 80%–85% of the reliable variance in the eight scales, leading eventually to the construction of two summary scores for physical and mental HRQOL. The use of summary scores provides the advantage of requiring fewer statistical comparisons in order to analyze SF-36 results, while not forfeiting the discrimination potential between physical and mental health status and outcomes. The two summary scores are usually normalized to a mean value of 50 and a standard deviation of 10 [20,21]. In a recent study in Greece, the SF-36 was validated and the results were comparable to those from studies in other European countries and the USA [22]. In a subsequent Greek study, the validity of the *Physical Component Summary (PCS) *and *Mental Component Summary (MCS) *scores was also established [23].

### The Greek Health Care System

The National Health System (ESY) in Greece was established in 1983 with an axiomatic aim to provide coverage for the entire population. It has evolved during the 1980s, and this is reflected by a substantial increase in public resources (e.g. beds, health centres and medical personnel) [24]. Meanwhile, the private sector, with a great tradition in Greece, managed to find ways to increase its market share by setting up diagnostic centres and investing in expensive medical technology [25]. Given also the increased number of physicians in Greece (physicians per 1000 individuals: 2.0 (1974), 2.8 (1983), 3.9 (1993) and 4.5 (2000)) [26], the Greek health care system continued to expand and became a typical example of a mixed (public-private) system [27].

The Greek health care system provides full coverage to the entire population, but at the same time has observed an increased use of the private sector. Access to all public services is free and there are no fees at the point of use, whereas the private sector requires out of pocket payments. Differences, between more and less prosperous insurance funds, concerning the extent and quality of services provided are evident. Health care services in Greece are basically provided by: a) the National Health System (public hospitals and health centres in rural and semi-urban areas) b) health units of Health Insurance Funds (health centres with salaried physicians or contracted physicians working in the private sector) and c) the private sector (hospitals, diagnostic centres, and private practitioners).

Health insurance funds are public schemes financed by employees, employers and the public budget. In Greece it is mandatory for the entire workforce (including their families) to be insured according to professional status, via one of 32 different health insurance funds [28]. It is worth mentioning that Greece spends 9.4% of its GDP on health, 59.1% of which is public expenditure coming from general taxation and social insurance contributions [29]. The rest is covered by private sources and mainly out of pocket payments, which confirms the noteworthy growth of the private sector [28]. Even after twenty years and a number of attempts to reform it, the Greek health care system remains fragmented in terms of coverage, and quite distanced from its principles of equity and efficiency [30].

## Methods

The study involved a stratified sample of residents of the broader Athens area, where approximately 35% of the Greek population lives. Institutionalized people were excluded. Participants were chosen proportionally to the population size, according to a three-staged sampling methodology. Specifically, in the first stage a random sample of 84 blocks of residences were selected according to information from the 1991 national census. In the second stage, households were selected from every block by systematic sampling. In the third stage, a participant (> 18 years) was chosen from every household by simple random sampling. Totally, 1007 out of 1426 candidates (response rate 70.6%) agreed to participate, constituting a representative sample of the population living in this particular area.

Participants were interviewed and the survey included the SF-36 and various health service utilization and socio-demographic questions. The necessary approval for carrying out this study has been provided by the Review Board of the Hellenic Open University. Physical and mental health summary scores, calculated and presented in previous work, were used as a proxy of health need. Principal Component Analysis was conducted to extract two components, which were subsequently rotated to an orthogonal simple structure using the Varimax method in order to facilitate comparisons with published results and simplify interpretation [23].

Health service utilization was measured by the visits: a) to public sector physicians within the last month b) to private sector physicians within the last month, c) to hospital emergency outpatient departments within the last year and by d) admissions to hospitals within the last year. Dependent variables were dichotomised to 0,1 values (0 → non use and 1 → use). Independent variables were grouped into three clusters, specifically i) demographic: age (continuous), gender (1 = male, 0 = women), marital status (dummy variable with reference category singles: 1 = married, 2 = divorced, 3 = widowed), ii) socio-economic: education level (1 = primary, 2 = secondary, 3 = lyceum, 4 = university), net monthly family income (continuous) and self-owned or rented residence (dummy variable with reference category residence freely provided) and iii) a proxy measure of need variable: SF-36 PCS and MCS scores. Only the variables demonstrating statistical significance (P < 0.05) were included.

Multivariate logistic regression models were implemented, one for each type of service, in order to determine predictors of health service utilization. Initially, access to health services was assessed and particularly the characteristics of those using the services compared to those not, and secondly the frequency of health service use, i.e. characteristics of those having used the services once compared to those using them more frequently. As Andersen and Newman [31], underline "*it makes considerable difference whether we are studying initial contact during a given period or whether we are studying the number of services received in a given period of time*". There is no clear perception on how frequent and non-frequent users should be distinguished, as they are defined arbitrarily and there is a lack of consensus in the literature [4].

Subsequently, we explored different predicting factors for the use of public, compared to private, services. Separate regression models were employed for primary and secondary services. The independent variables remained the same as in the analysis previously mentioned. The dependent variables were binary variables with 0, 1 values (0 → use of private services and 1 → use of public services). All statistical analyses were undertaken using SPSS v13.0.

## Results

### Health service utilization rates

Out of 1007 participants, 53.4% were women and the entire sample, with a mean age of 45 years, is classified into six age groups. Detailed socio-economic characteristics are provided in table 1. Four hundred fifty seven individuals had utilized at least one of the four types of health services, implying that 42.4% of the sample was considered as "users" (at least once) of public or private health services. Specifically, 26.5% of them used services affiliated to health insurance funds and 67.6% were one-time users. Accordingly, 13.9% of the participants had visited a private doctor and 64.9% of them were one-time users. The mean annual admission rates to emergency departments and to hospitals were 12.4% and 12.2% respectively, with 9.7% and 10.3% admitted at least once.

Upon initial investigation of the socio-economic characteristics of users and non-users (table 2), we observed that the users assessed that their general health was worse than that of non-users. At the same time their mental health, and even more so their physical health, was significantly lower. Women reported poorer physical and mental health compared to men (48.7% and 48.1% against 51.3% and 52.1%). Furthermore, an inverse relationship was witnessed between physical and mental health and age. Primary school graduates, the widowed and those with monthly family income less than 440 euros reported the poorest physical and mental health. Comparing physical and mental health of users and non-users (table 2) to that of the entire sample (table 1), users of all age groups -as it was expected- reported worse physical and mental health scores, with the exception of those aged 25–34 and 55–64. The same applies for all levels of education except in the case of high school graduates who report better mental health. In the case of marital status, it is the widowed (users) that report, once again, better mental health.

### Determinants of health service utilization

Given the four types of health service utilization designated in this study, we used regression models combining both public and private sector users, in order to determine the best predictors of utilization. Utilization of primary health services (table 3) appears to depend on demographic variables (13.8%), minimally on need variables (1%) and not at all on socio-economic factors. Women, elderly and those with worse physical health were more likely to use primary health services. Following this, separate models were implemented for public and private primary care users, and this resulted in various interesting observations.

Table 4 shows the regression model for last-month use of insurance fund primary services, which explained 18.2% of the variance. Demographic variables predicted 11.9% of the variation, socio-economic variables contributed with a further 2.3% to the explanation of health service utilization and health status, as measured by PCS and MCS scores, predicted a further 4.4%. More specifically, it appears that women utilized significantly more primary services, provided by health funds, than men. The elderly were 26.6% more likely to use these services, whereas wealthier people reported lower use. As for self-perceived health status, we observed that those with lower PCS scores were more likely to use health insurance services than individuals with higher PCS scores. Concerning visits to private physicians, within the context of primary health care, the regression model (table 3) explained only 13% of the variation. This figure is broken down to 7.9%, 3.0% and 2.1% variation explained by health status, socio-economic and demographic factors respectively. Women were more likely to visit private physicians. People with higher education were about 42% more likely to use primary health services from the private sector. Individuals with higher PCS scores were less likely to have consulted a private doctor within the last month.

Table 5 shows the regression model for the utilization of emergency department services and, in this case, only health status explained the variation. A trend was evident individuals with lower PCS and MCS summary scores reported higher use of emergency departments within the last year. The regression model for hospital admissions, shown also in table 4, predicted only 12.3% of the variation, with 10.1% predicted by health status and the remaining 2.2% by age. Specifically, age was related with admission and the elderly were more likely to be admitted, but after adjusting for health status, age was marginally not statistically significant. People with low PCS and MCS summary scores demonstrated a higher likelihood of having been admitted to a hospital within the last year.

### Frequency of health service utilization

In the previous analysis, regression models were implemented to distinguish between users and non-users in an attempt to examine possible socioeconomic barriers to initial care seeking. Another basic element, in this study, is the frequency of use for those making at least one visit, and the identification of predictors of the number of subsequent visits [31]. In the context of primary care, we classified people into two groups, those making one visit and those making more, and this was chosen because those who had made one visit to public and private sector physicians constituted 67.6% and 64.9% of the sample respectively. An inverse relationship was evident between family income and more than one visit to public sector physicians working for health insurance funds (table 6). Less wealthy and people with poorer mental health were associated with a higher likelihood of having made more than one visit. The regression model for subsequent visits to providers of the private sector resulted in statistical insignificance.

### Determinants of public health vs private health services utilization

The logistic regression model in table 7 focuses on the factors associated with utilization of public vs private primary health care services. Demographic variables explain only 7.8% of the total variance, and the addition of socioeconomic variables increases the explained proportion significantly to 16.9%. Married people were more likely to visit doctors affiliated to their insurance funds. More educated and wealthier people show higher likelihood to contact private doctors rather than their insurance fund doctors. The logistic regression model concerning hospitalization in public, compared to private, hospitals explains 44.8% of the total variance. Demographic, socioeconomic and health status variables contribute 19.5%, 21.6% and 3.7% respectively. Married, divorced and widowed are more likely (than singles), to be admitted to private (rather than to public) hospitals. The elderly show a significantly higher likelihood of being admitted to public hospitals. Indeed, the risk of being admitted to public hospitals increases 98% with age. Contrarily, as net monthly family income increases, people make more admissions to private hospitals. Finally people with higher MCS scores -better mental health- use the private hospitals more.

## Discussion

In this study we investigated the impact of demographic, socio-economic and need factors on the utilization of health services. The data showed that self-perceived health status -as a proxy measure of need- is the most important contributor to the utilization variance for three of the designated services (private physician, emergency departments and hospital admissions), a finding which is consistent with many previous studies [1-7]. Demographic variables such as age and gender were most strongly associated with visits to public (provided by health insurance funds) sector physicians. Socio-economic variables such as income and education did not have a statistically significant relationship with utilization, particularly in the case of secondary health services.

The results of the study seem to suggest the existence of equity in the use of primary health care services. Demographic and, to a lesser degree, need factors affected utilisation and no socio-economic gradient was apparent. Things were slightly different when the public and primary sectors were analysed separately, but once again inequity is not implied. Use of primary health services, provided by health insurance funds, was made according to demographic, socioeconomic and health care need variables. Women, elderly, less wealthy individuals and people with a lower physical health status visited their insurance fund physicians more. Demographic variables were the most important contributors and this may be explained by the fact that women reported higher consumption due to their increased awareness of health problems and symptoms when assessing their health status [6]. Furthermore age is a factor inversely linked to health, therefore elderly -a high-risk group from the aspect of health status and economic welfare- seek more public primary services, which are free at the point of use.

Utilisation of private services was also affected by socio-demographic and need factors. Women, well educated and those once again of lower physical health status were more likely to visit private providers. It was expected that economic factors like income would affect utilisation of private physicians, which involves out of pocket payments. However, this was not confirmed by our study as opposed to an earlier study in Italy [32], where a linear relationship between the level of income and private utilization was observed. A possible explanation, in our case, is the underestimation of income. One third of the sample had not reported income. Even people who did may have underestimated it because the Greek population is often reluctant to answer these kinds of questions.

Another noteworthy fact is that the independent variables in the regression model (table 4) explain only a low portion of utilisation of private primary services. Besides the underestimation of income mentioned previously, another reason could be the small proportion of users having visited a private physician (14.0%), since the sample comprises an overall healthy population. An interesting topic for future research is the users' satisfaction from public primary services and if quality variations actually directed them towards private physicians. An important implication, which will be discussed subsequently, is that low-income individuals use primary private services as well.

Visits to hospital emergency departments and hospital admissions were related to health care needs, and no socio-economic factor characterized the use of those types of care. As reported in an earlier study, hospital utilization and the volume of inpatient services were significantly influenced by medical needs [33] or as Andersen [34] explains, hospital services received in response to serious problems and conditions would be primarily explained by need and demographic characteristics. The small amount of variance explained here implies the coexistence of other factors (e.g. lifestyle) that could affect the utilization of these health services, and this itself is another issue for future research.

Upon examining the number of visits, no socio-economic influence was revealed. The poorer and those with worse mental health visited more frequently physicians linked to their health insurance fund. Other studies [3,4] have shown that people with higher education visited specialists more frequently or that they were more frequently referred to one. Socio-economic variations in the utilization of specialist services seem to be well-established in health systems in which referrals to specialists are made by primary physicians who play an important role in follow-up visits and hospital admissions. The structure of the Greek health system is different, primary health services concern mainly specialist services, which people choose freely without a referral.

Patients' preferences, awareness of their medical profile, availability of services and their expectations are important factors in seeking referred health care, mainly from specialists, in many European countries. Higher educated or wealthier individuals have different attitudes about the potential benefits, so they are more motivated to request specialist care [4]. In the Greek health system there is no observed inequality in access or frequent use, but patients' expectations, awareness of their condition and educational level consist basic factors in tackling a health problem within a complex mixed public-private health system.

After studying the different use of public/private services (table 7), a pro-rich inequality was observed. This does not contradict what has been previously mentioned about the use of private services only (table 4), where the effect of income is not evident, most likely because the respondents are homogenous (i.e. users of private services). Contrarily, income is important when the combined (public/private) users are studied. People better off in respect to education and income levels were more likely to use private health services. Results from another study in Greece [28] reported that the two higher income groups spend approximately the same amount of money as the others combined. This inequality becomes more severe when low-income people are forced to use needed health services from the private sector (because of the incomplete network of public primary health services, long waiting lists, "under the table" payments and low quality of provided services) burdening their limited family budget.

Low-income individuals have greater health care needs expressed by lower physical and mental summary scores, and further supported by research in western European countries showing that morbidity and mortality risks are higher in lower socioeconomic groups [35]. Although it seems that low-income individuals generally use health services, it is apparent that they are not exclusive users of the public services, but they are often forced to use private health services as well. This implies inequity in the distribution of care since the consumption of private health services is not limited to the higher incomes, but is extended to the lower ones as well, thus giving rise to issues of horizontal and vertical inequity. A possible explanation could be the inadequate public financing. Greece has the lowest percentage of public health expenditures among the EU countries. According to OECD data in 2002 [29], total health expenditures per capita were 1814$, of which public health expenditures per capita were only 960$. On the contrary, private expenditures are the highest among the EU countries, and this means that the income, in all socio-economic classes, is burdened for the use of health services.

Another possible explanation is the structural problems of the system. The large number of health insurance funds and the different range of health services they cover is the most typical characteristic in this case. More specifically, wealthier funds cover a large range of services, provide a better set of inpatient services or, in many instances, offer reimbursement when individuals purchase from private providers. Often, people insured by the most prosperous health insurance funds (approximately 10% of the insured population) are covered, to a large extent, for hospitalization in prestigious private hospitals, for all illnesses, specialized operations and examinations [28]. This unequal distribution of provided health services, within the public sector, constitutes a structural weakness of the Greek health system, which the private sector exploits.

In spite of social insurance coverage of Greek citizens, the use of private services is extensive throughout the country. This could be attributed also to the absence of the "family doctor" in Greece, and the inability to select the desired physician in primary and secondary health care. The absence of the family doctor affects the delivery of care, access and the referrals to the health system. This results in patients accessing secondary care based on their own initiative. The adoption of a gate keeping system could result in a link between primary and secondary care, and an effective patient transfer. Moreover it could give emphasis to issues such as prevention and over-consumption (especially of hospital care), which pose a great burden on the health system [36]. On the other hand, insurance funds covering people of low income are either incapable of fulfilling their needs, or they provide lower quality health care, in conjunction to long waiting lists, all of which affect the degree of satisfaction. Overall, a significant personal cost is created for the users, who are forced to turn to the private sector and spend a large part of their income. This implies that a more complete and satisfactory network of services could result in lower use of the private sector.

Finally, two limitations should be briefly underlined. First, this study concerned the broader Athens area, the capital of Greece, where medical personnel is more experienced and better equipped technologically, and consequently more specialised health services are available. The heterogeneous dispersion of resources in relation to the population throughout the country reflects an unequal availability of health services, which is expected to be more intense in rural areas. So the first restriction reflects lack of data on the pattern of use of the rural population. Another limitation, reported also in a study by Morris [2], was the fact that utilization measures were zero-one variables for four defined types of use and there was no information on the quality of provided services.

## Conclusion

This study has demonstrated a positive relationship between health need and the utilisation of health services under a mixed public-private funded health care system. Health need, defined by self-perceived health status, was the most important determinant for visiting public or private sector physicians, emergency departments and admissions to the hospitals. Moreover, health need and low income were the main factors influencing subsequent visits to public sector physicians. Concerning the use of public vs private services, we observed that socio-economic characteristics of individuals were the main determinants. People with higher education and income levels used more private sector services, although they were not the exclusive users, since low-income groups used private services as well. In Greece, it seems that access into the health system is relatively easy. However the aim is to access a complete, uniform and satisfying public health system with respect to the quality and the extent of provided services. Despite 25 years of Greek NHS reform, this target has not yet been achieved.

## Competing interests

The author(s) declare that they have no competing interests.

## Authors' contributions

EP has participated in the design of the study, the statistical analysis, the interpretation of the results and drafted the manuscript. DN conceived the study, interpreted the results and made refinements of the manuscript. Both authors have read and approved the final manuscript.

## Pre-publication history

The pre-publication history for this paper can be accessed here:



## Figures and Tables

**Table 1:** Socio-demographic characteristics of the sample population and mean physical and mental summary scores

**Sex**	**n**	**%**	**PCS (CI)**	**MCS (CI)**
Male	469	46.6	51.9 (50.5–52.2)	52.1 (51.3–52.9)
Female	538	53.4	48.8 (47.8–49.6)	48.1 (47.2–49.0)
				
**Age Group**				
18–24	144	14.3	55.5 (54.7–56.2)	50.4 (48.9–51.8)
25–34	198	19.7	54.1 (53.2–55.1)	51.8 (50.6–53.0)
35–44	188	18.6	52.4 (51.3–53.5)	48.8 (47.1–50.6)
45–54	155	15.4	48.8 (47.2–50.5)	49.9 (48.2–51.7)
55–54	155	15.3	47.7 (46.1–49.2)	49.9 (48.2–51.7)
65+	167	16.1	40.6 (38.8–42.2)	48.2 (46.5–49.8)
				
**Education**				
Primary	201	20.0	43.5 (41.8–45.2)	46.2 (44.5–47.8)
High school	116	11.5	50.3 (48.3–52.2)	48.6 (46.6–50.6)
Lyceum	424	42.1	51.9 (51.1–52.6)	50.6 (49.7–51.4)
University	259	25.8	51.7 (50.6–52.7)	52.5 (51.4–53.5)
				
**Marital status**				
Single	278	27.6	54.2 (53.3–55.1)	50.7 (49.7–51.8)
Married	604	60.0	49.2 (48.5–50.0)	50.4 (49.6–51.2)
Divorced	46	4.6	50.3 (47.7–53.1)	50.1 (46.8–53.3)
Widowed	74	7.3	39.6 (36.4–42.8)	43.5 (40.6–43.5)
				
**Income (euros)**				
< 440.00	35	3.5	37.3 (33.3–41.3)	40.6 (36.4–44.8)
440.01–880.4	227	22.5	47.3 (45.8–48.8)	49.1 (47.7–50.4)
883.34–1320.6	220	21.8	50.1 (48.7–51.5)	50.3 (48.9–51.7)
1323.5–1760.82	149	14.8	51.4 (50.2–52.7)	52.0 (50.6–53.3)
1763.75–2201.00 83	8.2	52.6 (51.1–54.2)	49.6 (47.6–51.6)	
2204.00+	53	5.3	53.3 (51.3–55.3)	52.1 (49.5–54.6)
Missing	241	23.9	51.6 (50.5–52.7)	50.2 (49.0–51.5)

**Table 2:** Mean physical and mental summary scores for users & non-users stratified by socioeconomic characteristics

	**PCS (CI)**	**MCS (CI)**		
**non-users**	52.8 (52.1–53.5)	50.9 (50.2–51.7)		
**users**	46.6 (45.6–47.5)	48.2 (47.7–49.8)		
				
	**Users**		**Non-users**	
**Sex**	**PCS (CI)**	**MCS (CI)**	**PCS (CI)**	**MCS (CI)**
		
Men	47.3 (45.7–48.8)	50.5 (49.1–52.0)	53.9 (53.1–54.7)	53.1 (52.3–53.9)
Women	46.1 (44.8–47.4)	47.6 (46.2–49.5)	51.6 (50.4–52.7)	48.6 (47.4–49.7)
				
**Age**				
18–24	54.0 (52.6–55.3)	47.0 (45.1–50.6)	56.4 (55.5–57.2)	51.9 (50.3–53.6)
25–34	52.0 (50.2–53.8)	52.4 (50.2–54.7)	55.5 (55.4–56.5)	51.4 (50.1–52.7)
35–44	49.4 (47.0–51.7)	49.1 (46.4–51.9)	54.0 (52.9–55.1)	50.8 (49.2–52.4)
45–54	44.5 (41.9–47.1)	45.7 (42.7–48.8)	52.5 (50.6–54.3)	51.5 (49.7–53.3)
55–64	45.5 (43.3–47.6)	50.3 (48.0–52.7)	50.6 (48.4–52.8)	49.3 (46.7–51.9)
65+	39.1 (36.9–41.2)	47.1 (44.9–49.2)	43.3 (40.2–46.4)	49.8 (47.2–52.5)
				
**Educational level**				
Primary	40.1 (38.1–42.1)	45.2 (42.9–47.5)	48.0 (45.3–50.7)	47.4 (45.1–49.8)
High school 47.7 (44.6–50.8)	49.9 (46.9–52.8)	52.3 (49.9–54.6)	47.6 (44.9–50.3)	
Lyceum	49.1 (47.8–50.4)	49.3 (47.8–50.8)	54.1 (53.3 54.9)	51.6 (50.6–52.6)
University	48.5 (46.4–50.6)	51.4 (49.3–53.4)	53.7 (52.7–54.7)	53.2 (52.1–54.2)
				
**Marital status**				
Single	51.8 (50.1–53.4)	48.9 (46.9–50.9)	55.7 (54.8–56.6)	51.9 (50.8–53.1)
Married	45.8 (44.7–47.0)	49.9 (48.6–51.2)	52.1 (51.2–53.1)	50.9 (50.9–49.9)
Divorced	49.1 (43.9–54.2)	44.7 (38.5–50.8)	51.3 (48.3–54.4)	54.1 (51.5–56.7)
Widowed	37.1 (33.0–41.1)	43.8 (39.7–47.8)	44.1 (39.0–49.2)	42.9 (38.2–47.6)
				
**Income (euros)**				
< 440.00	36.3 (32.0–40.5)	40.2 (35.1–45.2)	39.4 (29.9–48.9)	41.5 (32.6–50.3)
440.01–880.4	43.9 (41.7–46.0)	47.6 (45.4–47.9)	50.9 (49.0–52.8)	50.7 (49.1–52.2)
883.34–1320.6	45.0 (42.5–47.4)	49.7 (47.2–52.1)	53.4 (52.1–54.7)	50.7 (49.0–52.4)
1323.5–1760.82	49.6 (47.5–51.6)	51.6 (49.0–54.1)	52.8 (51.3–54.3)	52.4 (51.0–53.8)
1763.75–2201.00	49.6 (47.2–51.9)	49.8 (46.7–52.3)	56.1 (54.5–57.6)	49.4 (46.7–52.1)
2204.00+	50.6 (46.6–54.5)	50.5 (45.8–55.1)	55.6 (54.3–56.8)	53.4 (50.7–56.1)
Missing	49.3 (47.3–51.3)	48.8 (46.7–51.0)	53.4 (52.1–54.6)	51.2 (49.8–52.6)

**Table 3:** Utilization of primary health services

**Variables**	**B**	**Exp(B)**	**CI(95%)**
Sex	-.497	.608	.445–.832
Age	.231	1.260	1.106–1.435
Marital Status	_	_	_
Partial R^2^	.138		
			
Education	_	_	_
Self-owned			
Residence	_	_	_
Income	_	_	_
Partial R^2^	_		
			
PCS	-.049	.952	.945–969
MCS	_	_	_
Partial R^2^	.010		
			
Nagelkerke R^2^	0.148		

**Table 4:** Utilization of public and private sector physician services

	**Public sector**			**Private Sector**		
**Variables**	**B**	**Exp(B)**	**CI(95%)**	**B**	**Exp(B)**	**CI(95%)**
Sex	-.387	.679	.493–.934	-.670	.512	.344–762
Age	.236	1.266	1.121–1.431	_	_	_
Marital Status	_	_	_	_	_	_
Partial R^2^	.119			.021		
Education	_	_	_	.353	1.423	1.175–1.724
Self-owned						
Residence	_	_	_	_	_	_
Income	-.078	.925	.871–.983	_	_	_
Partial R^2^	.023			.030		
PCS	-.041	.960	.945–976	-.048	.953	.936–.970
MCS	_	_	_	_	_	_
Partial R^2^	.044			.079		
Nagelkerke R^2^	0.182			0.130		

**Table 5:** Utilisation of Emergency Departments and Hospital Admissions

	**Utilisation of Emergency Depart.**			**Admissions to Hospital**		
**Variables**	**B**	**Exp(B)**	**CI(95%)**	**B**	**Exp(B)**	**CI(95%)**
Sex	_	_	_	_	_	_
Age	_	_	_	_	_	_
Marital Status	_	_	_	_	_	_
Partial R^2^					.022	
						
Education	_	_	_	_	_	_
Self owned residence	_	_	_	_	_	_
Income	_	_	_	_	_	_
Partial R^2^						
						
PCS	-.041	.960	.944–.976	-.058	.943	.925–.962
MCS	-.034	.967	.950–.983	-.026	.974	.957–.992
Partial R^2^				.101		
Nagelkerke R^2^	0.071			0.123		

**Table 6:** Frequency of visits to public-sector physicians

**Variables**	**B**	**Exp(B)**	**CI(95%)**
Sex	_	_	_
Age			
Marital Status	_	_	_
Partial R^2^	_		
			
Education	_	_	_
Self-owned residence	_	_	_
Income	-.274	.760	.601–.962
Partial R^2^	.076		
			
PCS	_	_	_
MCS	-.054	.948	.917–.980
Partial R^2^	.071		
			
Nagelkerke R^2^	.147		

**Table 7:** Utilisation of public vs private services

	**Primary**			**Secondary**		
**Variables**	**B**	**Exp(B)**	**CI(95%)**	**B**	**Exp(B)**	**CI(95%)**
Sex	_	_	_	_	_	_
Age	_	_	_	.686	1.986	1.260–3.131
Married	.727	2.069	1.136–3.768	-2.004	.135	.024–.744
Divorced	_	_	_	-4.081	.017	.001–.396
Widowed	_	_	_	-3.091	.045	.002–.925
Partial R^2^	.078			.195		
						
Education	-.440	.644	.479–.866	_	_	_
Self owned residence	_	_	_	_	_	_
Income	-.132	.877	.791–.971	-.207	.813	.673–.982
Partial R^2^	.091			.216		
						
PCS	_	_	_	_	_	_
MCS	_	_	_	-1.334	.263	.072–.985
Partial R^2^				.037		
						
Nagelkerke R^2^	0.169			0.448		
